# Biological Skin Substitutes in Pressure Ulcers: High-Purity Type I Collagen-Based Versus Amnion/Chorion Membrane

**DOI:** 10.7759/cureus.90956

**Published:** 2025-08-25

**Authors:** Naveen Narayan, Yashas H Ramegowda, Divakara S Raghupathi, Shivannaiah Chethan, Suhas Gowda

**Affiliations:** 1 Plastic Reconstructive and Aesthetic Surgery, Adichunchanagiri Institute of Medical Sciences, Bala Gangadharanatha Nagara, IND; 2 General Surgery, Akash Medical College, Bangalore, IND; 3 General Surgery, Adichunchanagiri Institute of Medical Sciences, Bala Gangadharanatha Nagara, IND

**Keywords:** bed sores, biological skin substitute, dehydrated human amnion/chorion membrane, helicoll®, high-purity type i collagen, pressure ulcer, randomized controlled trial, wound healing

## Abstract

Background: Pressure ulcers (PUs) present a therapeutic challenge in bedridden or neurologically impaired patients and represent a significant healthcare burden, affecting millions of patients worldwide. Current standard treatment approaches often fall short in achieving optimal healing outcomes, necessitating the development of advanced therapeutic interventions. Biological skin substitutes - high‐purity type I collagen (HPTC) and dehydrated human amnion/chorion membrane (dHACM) - have emerged as promising therapeutic alternatives to traditional wound care approaches. This study compares their effectiveness in treating stage III-IV PUs.

Methods: This prospective, randomized, controlled, open-label, two parallel group, single-centre clinical trial was conducted at Adichunchanagiri Institute of Medical Sciences, a tertiary care hospital in India, specializing in wound care and reconstructive surgery. We conducted a parallel group, open-label, randomized controlled trial with 80 patients (n = 40 per group) with stage III or IV PUs randomized 1:1 to receive either HPTC (Helicoll^®^) or dHACM, applied alongside standard wound care. The primary endpoint was complete ulcer closure by seven weeks, including a one-week follow-up. Secondary outcomes included time to closure, vascularity infiltration, reduction in wound area, number of repeated applications, adverse events, scar improvement, structural stability, and patient satisfaction. Statistical analysis was performed using appropriate parametric and non-parametric tests.

Results: The HPTCgroup demonstrated superior outcomes with 30 patients (75%) having complete wound closure compared to 25 patients (62.5%) in the dHACM group (p = 0.234). Mean percentage wound closure was 78.5% ± 18.2% for HPTC versus 65.1% ± 9.8% for dHACM (p < 0.05). HPTCshowed enhanced vascular infiltration (Grade 3: 31 patients (77.5%) vs. eight patients (20%) (p < 0.001)), improved neo-epithelialization (Grade 3: 28 patients (70%) vs. nine patients (22.5%) (p < 0.001)), and increased fibroblast activity (Grade 3: 33 patients (82.5%) vs. seven patients (17.5%) (p < 0.001)) at day 5. The HPTCgroup required fewer reapplications (0.85 ± 0.92 vs. 1.15 ± 0.87, p = 0.127). No serious adverse events were reported in either group. Adverse events were minor and similar between groups. The HPTC group demonstrated superior outcomes with 29 patients (72.5%) showing improved patient satisfaction compared to 17 patients (42.5%) in the dHACM group.

Conclusion: Helicoll^® ^(HPTC) showed numerically superior healing rates, improved tissue regeneration parameters, required fewer reapplications with better structural stability of the healed wound, improved patient satisfaction outcomes compared to dHACM, suggesting potential clinical and economic advantages. These findings support the use of Helicoll^®^ as an effective therapeutic option for PU management.

## Introduction

Pressure ulcers (PUs), also known as decubitus ulcers or bedsores, represent one of the most challenging complications in healthcare settings, particularly affecting immobilized patients, elderly individuals, and those with chronic medical conditions, in long-term care facilities, and represent a significant healthcare challenge affecting millions of patients worldwide [1]. The prevalence of PUs ranges from 10% to 18% in acute care settings and can be as high as 28% in long-term care facilities [2]. The global prevalence ranges from 8.8% to 53% across different healthcare settings, with associated annual treatment costs exceeding $11 billion in the United States alone [3,4].

The pathophysiology of PUs involves a complex cascade of events initiated by sustained pressure that exceeds capillary perfusion pressure, typically 32 mmHg. These wounds develop as a result of prolonged pressure, shear forces, and friction that compromise local tissue perfusion, leading to ischemia, inflammatory responses, necrosis, and subsequent ulceration [5,6]. Thus, the development of PUs involves complex interactions between mechanical factors, tissue tolerance, and patient-specific risk factors, including advanced age, malnutrition, diabetes mellitus, vascular disease, and compromised mobility [7]. The National Pressure Ulcer Advisory Panel classification system categorizes PUs into four stages based on tissue involvement, from superficial skin changes (stage I) to full-thickness tissue loss with exposed bone, tendon, or muscle (stage IV) [8].

Management of PUs is multifaceted. Standard care, including repositioning, offloading (pressure redistribution), debridement, infection control, and moist wound environment, while essential, often proves insufficient for achieving timely wound closure, particularly in chronic, non-healing ulcers, and often yields suboptimal outcomes [9,10]. Moreover, PUs are associated with increased morbidity, prolonged hospital stays, and reduced quality of life, making effective treatment strategies a clinical priority [11]. The management of PUs presents unique challenges compared to other chronic wounds due to their location, patient comorbidities, and tendency for recurrence. The anatomical sites commonly affected by PUs, including sacral, gluteal, heel, and lateral malleolus regions, require specialized treatment approaches that consider both local wound factors and systemic patient conditions [12]. Furthermore, the economic burden associated with prolonged treatment periods and potential complications necessitates the evaluation of cost-effective therapeutic interventions.

The evolution of wound care has witnessed significant advances in biological therapies, with skin substitutes emerging as promising therapeutic modalities. These products aim to provide a biological scaffold that supports cellular migration, angiogenesis, and tissue regeneration while addressing the fundamental deficiencies in chronic wound environments [13]. Acellular dermal substitutes may be composed of naturally occurring polymers such as collagen, elastin or hyaluronic acid, synthetic polymers, porcine dermis, or de-epithelialized cadaveric skin [14]. Biological skin substitutes may augment regeneration via the provision of extracellular matrix scaffolding, growth factors, and anti-inflammatory mediators, along with providing structural support and promoting angiogenesis [8]. Among the available options, high-purity type I collagen-based skin substitutes (HPTCs) and dehydrated human amnion/chorion membranes (dHACMs) have gained considerable attention due to their unique biological properties and clinical efficacy.

Collagen, the most abundant protein in the human body, serves as a natural scaffold for tissue regeneration and plays a crucial role in all phases of wound healing [1]. It provides structural support, facilitates cellular adhesion and migration, and serves as a haemostatic agent [15]. HPTCs, such as Helicoll®, are derived from bovine or porcine sources and processed to maintain bioactivity while ensuring safety and sterility [16]. They have demonstrated efficacy in various wound types, including diabetic foot ulcers and chronic wounds, showing superior healing compared to standard care and, in some cases, to amnion-derived products [17]. They have emerged as promising therapeutic options due to their biocompatibility, biodegradability, low immunogenicity, and ability to support cellular migration and proliferation [18]. Collagen scaffolds stimulate fibroblast activity, angiogenesis, and granulation tissue formation.

dHACMs represent another innovative approach to wound management. These allografts are derived from the innermost layer of the placenta and retain growth factors, cytokines, and extracellular matrix components that promote healing [19].

Some randomized controlled trials have compared these biological skin substitutes in diabetic foot ulcer populations [20,21,22,23]. Skin substitutes are ideal for non-healing or difficult-to-heal wounds, including vascular insufficiency ulcers, diabetic neuropathic ulcers, and PUs [24]. A recent study demonstrated that HPTC achieved a mean percentage area reduction of 83.9% by four weeks post-randomization, while other studies have shown varying results depending on wound characteristics and patient populations [25,26,27]. However, comparative data specifically focusing on PUs remain limited, creating a knowledge gap that warrants investigation. Despite the growing body of literature supporting the use of biological skin substitutes in wound care, direct comparative studies evaluating their efficacy in PU treatment remain limited. Previous research has primarily focused on diabetic foot ulcers and venous leg ulcers, with scarce studies specifically addressing PUs [28]. This knowledge gap underscores the need for well-designed clinical trials to establish the relative efficacy of these advanced wound care products in PU management.

Given the growing evidence supporting biological skin substitutes in wound care and the specific challenges associated with PU management, this study aimed to compare the clinical efficacy, safety, and practical considerations of HPTC versus dHACM in the treatment of PUs. The findings from this randomized controlled trial will contribute to evidence-based treatment protocols and inform clinical decision-making in PU management, especially stage III and IV PUs.

## Materials and methods

This prospective, randomized, controlled, open-label, two-parallel-group, single-centre clinical trial was conducted at Adichunchanagiri Institute of Medical Sciences, a tertiary care hospital in India, specializing in wound care and reconstructive surgery. The study protocol was approved by the Institutional Ethics Committee (approval number: AIMS/IEC/012/2025) and registered with ClinicalTrials.gov (NCT06853210). All procedures were performed in accordance with the Declaration of Helsinki and Good Clinical Practice guidelines. All participants or their guardians provided informed consent.

Eligible participants were adults aged 18-80 years with stage III or IV PUs as defined by the National Pressure Ulcer Advisory Panel. Inclusion criteria included (a) presence of a PU measuring between 2 cm² and 25 cm², (b) ulcer duration of at least four weeks prior to enrolment, (c) adequate blood supply to the affected area confirmed by clinical assessment and triphasic Doppler studies, (d) diabetes mellitus with controlled glycaemic status (HbA1c <10%), (e) compliance with off-loading and pressure redistribution measures, (f) ability to attend required study visits, and (g) willingness to participate in the informed consent process.

Exclusion criteria included (a) life expectancy less than six months, (b) presence of active infection or osteomyelitis in target ulcer requiring systemic antibiotics, (c) known allergy to components of HPTC or dHACM, (d) severe malnutrition (albumin <2.5 g/dL), (e) autoimmune or connective tissue disorders, (f) pregnancy or breastfeeding, and (g) history of immunosuppressive therapy, malignancy, systemic steroids, or uncontrolled diabetes (HbA1c >10%).

Randomization and blinding

Eligible participants were randomly assigned in a 1:1 ratio to receive either HPTC or dHACM using a computer-generated randomization sequence. Randomization was stratified by ulcer location (sacral, gluteal, heel, lateral malleolus, elbow) to ensure balanced distribution. Due to the nature of the interventions, blinding of participants and investigators was not feasible, resulting in an open-label design. However, outcome assessment was done by a blinded wound care assessor during wound measurements and photography. Moreover, the pathologists doing histopathological analysis were blinded.

Interventions

Group A patients (n = 40) received HPTC-based skin substitute (Helicoll®, Encoll Corporation, USA) applied directly to the clean wound bed after debridement. The product was sized to cover the entire wound area with a 2-3 mm overlap onto healthy surrounding tissue. Secondary dressing consisted of a non-adherent contact layer, an absorbent pad, and an adhesive border dressing with a compression bandage as required.

Group B patients (n = 40) received dHACM (Amchoplast, LifeCell International Pvt. Ltd.) rehydrated according to manufacturer instructions and applied to the clean wound bed. A similar secondary dressing protocol was followed as in Group A.

Standard care for both groups included weekly debridement (if required), infection control measures, offloading by repositioning and pressure relief mattresses, nutritional optimization, diabetic management and comorbidity optimization, and patient and caregiver education.

Outcome measures

The primary outcome measure included the percentage wound area reduction from week 1 through week 6, plus a one-week follow-up, measured manually with digital photography.

Secondary outcome measures were infiltration of vascularity in the ulcer bed, proportion of subjects to obtain complete closure, time to achieve complete wound closure, mean number of repeated application, and incidence of adverse events. Infiltration of vascularity in the ulcer bed on day 5 of application - vascularity assessment will be done using biopsy on day 0 of application to be compared with day 5 after application of HPTC/dHACM. Before application and on the fifth day post-application of either HPTC or dHACM, a 2 mm punch biopsy was obtained from the wound edge extending into the wound bed under local anaesthesia (2% lidocaine without epinephrine). Biopsy samples were immediately fixed in 10% neutral buffered formalin for 24 hours, processed through graded alcohols, and embedded in paraffin blocks. Serial sections of 4 μm thickness were prepared and stained with haematoxylin and eosin (H&E) for general morphology, Masson's trichrome for collagen assessment, CD31 immunohistochemistry for capillary density evaluation, and α-SMA immunohistochemistry for fibroblast activity. Histological parameters that were evaluated included vascular infiltration, neo-epithelialization, fibroblast activity, capillary density, inflammatory response, and collagen deposition (Table 1).

**Table 1:** Histological parameters evaluated to assess infiltration of vascularity in the ulcer bed on day 0 and day 5 after application

Sl. No.	Parameter	Measurement tool	Criteria	Score
1	Vascular Infiltration	Assessed by counting new blood vessels (0-3 scale)	Minimal vascular ingrowth (<5 vessels/hpf)	0
			Mild infiltration (5-10 vessels/hpf)	1
			Moderate infiltration (11-20 vessels/hpf)	2
			Abundant infiltration (>20 vessels/hpf)	3
2	Neo-epithelialization	Measured as epithelial migration distance from wound edge (0-3 scale)	No epithelial migration	0
			Minimal migration (<25% wound coverage)	1
			Moderate migration (25-75% coverage)	2
			Extensive migration (>75% coverage)	3
3	Fibroblast Activity	Quantified by counting α-SMA positive fibroblasts per HPF and assessment of fibroblast morphology (0-3 scale)	Sparse, inactive fibroblasts	0
			Moderate cellularity, minimal matrix production	1
			High cellularity, active-matrix synthesis	2
			Very high activity with extensive matrix deposition	3
4	Capillary Density	Evaluated using CD31 staining, counted as vessels per mm² of tissue		
5	Inflammatory Response	Graded semi-quantitatively (0-3 scale)	Minimal inflammatory infiltrate	0
			Mild chronic inflammation	1
			Moderate mixed inflammation	2
			Severe acute inflammation	3
6	Collagen Deposition	Assessed using Masson's Trichrome staining (0-3 scale)	Minimal collagen matrix	0
			Loose, immature collagen	1
			Moderate organized collagen	2
			Dense, mature collagen architecture	3

Second, the proportion of subjects to obtain complete closure, defined as 100% epithelialization with no drainage, of the target ulcer was determined by the proportion of subjects that obtained complete closure over the six-week treatment period. Third, the wound size progression over time was assessed. Next, the mean number of repeated applications of the HPTC and dHACM used to obtain wound closure over six weeks was noted. Lastly, the incidence of adverse events related to the intervention, such as infection, allergic reactions, if any, was noted over six weeks of the study period.

Other pre-specified outcome measures included patient treatment satisfaction assessed by using a Likert scale measured with a score range from 1 to 5, wherein 1 = "extremely unsatisfied" to 5 = "extremely satisfied.” Finally, healed wound appearance assessment was determined using Manchester Scar Scale - the resultant new skin is assessed and documented at each visit using the Manchester Scar Scale, assessing colour, finish, contour, distortion, and texture with values given from 1 to 4, wherein 1 denotes excellent and 4 means poor. This was done at the follow-up period on the seventh week.

Sample size and statistical analysis

The sample size calculation was based on the expected difference in wound closure rates between groups. Assuming an 85% closure rate in the HPTC group versus 65% in the dHACM group, with 80% power and 5% significance level, 38 patients per group were required. To account for a 10% dropout rate, 40 patients per group were enrolled.

Statistical analysis was performed using IBM SPSS Statistics for Windows, version 26.0 (IBM Corp., Armonk, NY). Continuous variables were presented as mean ± standard deviation or median (interquartile range) based on distribution normality assessed by the Kolmogorov-Smirnov test. Categorical variables were presented as frequencies and percentages.

Between-group comparisons for continuous variables were performed using an independent t-test or a Mann-Whitney U test, as appropriate. Chi-square test or Fisher's exact test was used for categorical variables. Time-to-event analysis was performed using Kaplan-Meier survival curves with the log-rank test. Repeated-measures ANOVA was used to analyze wound size changes over time.

Statistical significance was set at p < 0.05. All analyses were performed on an intention-to-treat basis with last observation carried forward for missing data.

Data collection and follow-up

Demographic data, medical history, comorbidities, and baseline wound characteristics were recorded. Laboratory parameters, including hemoglobin, albumin, and glycated hemoglobin (HbA1c), were obtained at baseline and follow-up visits.

Patients were evaluated at baseline, day 5, day 14, day 21, day 28, day 35, and day 42. At each visit, wound measurements were performed using standardized digital planimetry, with wound area calculated in cm². Histopathology examination results were recorded on baseline day 0 and day 5 after application. Digital photographs were taken for documentation and independent assessment. Complete wound closure was defined as 100% epithelialization without drainage or dressing requirements, confirmed at two consecutive visits. Any adverse events, if they occurred, were duly noted and notified. Scar quality and durability were assessed at each visit, with patient satisfaction assessed at the seventh week, during the one-week follow-up.

## Results

**Figure 1:**
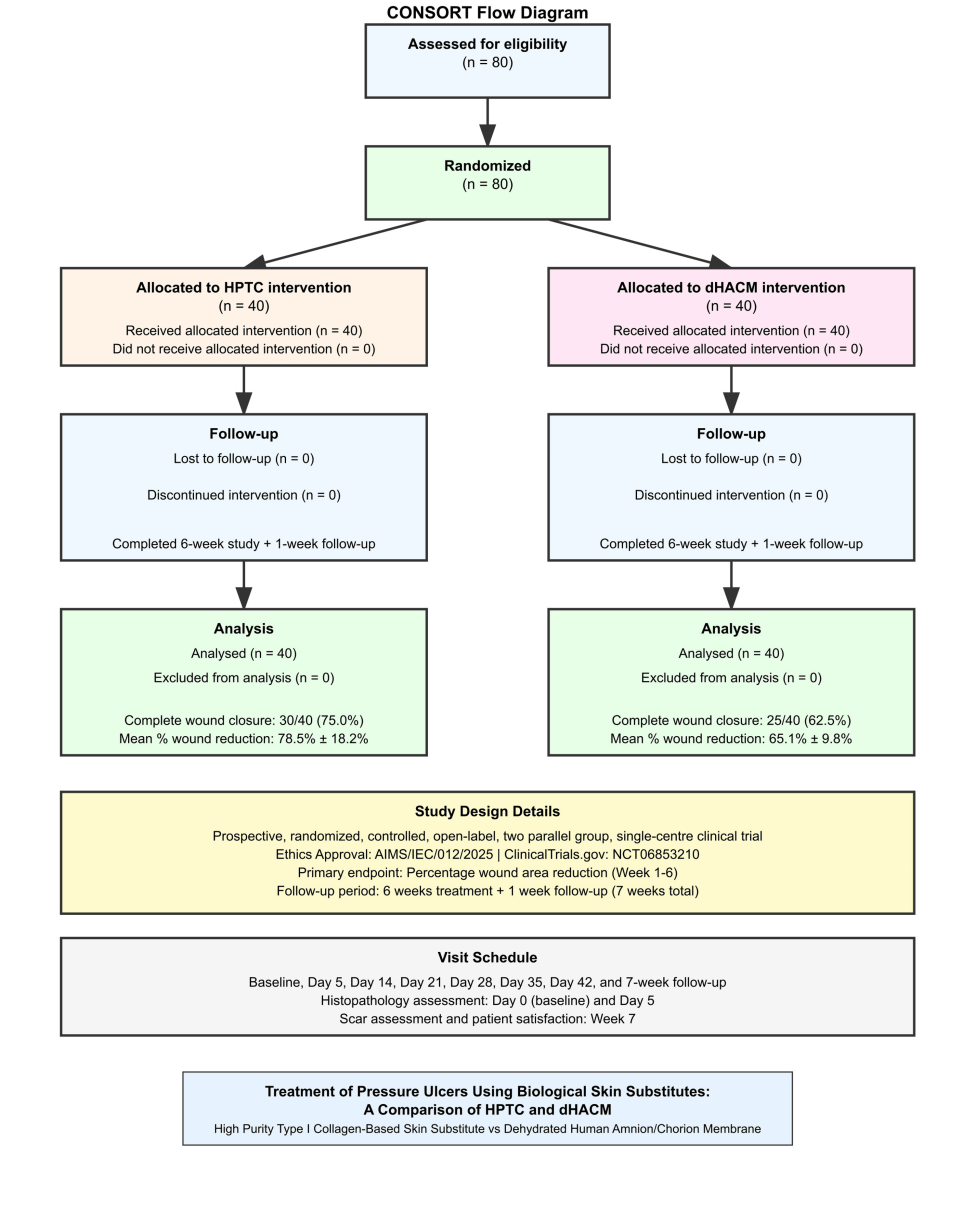
CONSORT diagram describing the flow of the participants

A total of 80 patients with stage III or IV PUs were enrolled and randomized to receive either HPTC (n = 40) or dHACM (n = 40) treatment. All patients completed the six-week study plus one-week follow-up (seven-week period) with no dropouts recorded. The CONSORT diagram describing the flow of the participants is shown in Fig. 1.

Baseline demographics and clinical characteristics were comparable between groups and are summarized in Table 2. The mean age was 63.5 ± 9.8 years in the HPTC group and 66.2 ± 11.2 years in the dHACM group (p = 0.234). Gender distribution showed 28 (70%) males in the HPTC group versus 22 (55%) males in the dHACM group (p = 0.123). The remainder were females. Comorbidities, diabetes mellitus, and hypertension were in equal distribution across groups, the difference being statistically insignificant. Ulcer duration in the HPTC group was 2.1 ± 1.1 months and 2.3 ± 1.2 months in the dHACM group (p = 0.456). Baseline ulcer size was 14.9 ± 2.1 cm^2^ and 15.0 ± 1.8 cm^2^ in HPTC and dHACM groups (p = 0.821). Most ulcers were stage III (22 patients (55%) in the HPTC group vs. 21 patients (52.5%) in the dHACM group).

**Table 2:** Baseline patient characteristics

Variable	HPTC (n = 40)	dHACM (n = 40)	p-value
1. Age (years), mean ± SD	63.5 ± 9.8	66.2 ± 11.2	0.234
2. Gender, n (%)			
Male	28 (70%)	22 (55%)	0.123
Female	12 (30%)	18 (45%)	
3. Diabetes mellitus, n (%)	16 (40%)	16 (40%)	1.000
4. Hypertension, n (%)	22 (55%)	20 (50%)	0.651
5. Ulcer duration (months), mean ± SD	2.1 ± 1.1	2.3 ± 1.2	0.456
6. Baseline ulcer size (cm²), mean ± SD	14.9 ± 2.1	15.0 ± 1.8	0.821

Ulcer location distribution

In this comparative study of pressure sore treatment, the distribution of ulcer locations was similar between the HPTC and dHACM treatment groups. The gluteal region was the most common site for PUs in both groups, accounting for 16 cases (40.0%) in each treatment arm. The sacral region was the second most frequent location, with 11 cases (27.5%) in the HPTC group and 12 cases (30.0%) in the dHACM group. Ulcers in the elbow region were the least common, with two cases (5.0%) observed in each treatment group, demonstrating nearly identical anatomical distribution patterns between the two therapeutic approaches (Table 3).

**Table 3:** Ulcer location distribution

Ulcer location	HPTC n (%)	dHACM n (%)
Gluteal region	16 (40.0%)	16 (40.0%)
Sacral region	11 (27.5%)	12 (30.0%)
Lateral malleolus	5 (12.5%)	6 (15.0%)
Heel	6 (15.0%)	4 (10.0%)
Elbow	2 (5.0%)	2 (5.0%)

All patients had triphasic Doppler studies confirming adequate arterial perfusion. The baseline characteristics were well-balanced between groups, indicating successful randomization.

Complete wound closure rates

At seven weeks of follow-up, complete wound closure was achieved in 30 patients (75.0%) in the HPTC group compared to 25 (62.5%) in the dHACM group. While the HPTC group showed numerically superior healing rates, this difference did not reach statistical significance (p = 0.234, Chi-square test).

Percentage wound area reduction

**Figure 2:**
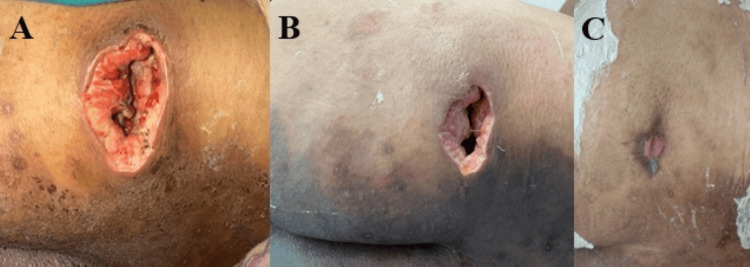
Left gluteal pressure sore immediately after debridement (A); Post Helicoll® application, on day 5 of punch biopsy, showing relatively contracted wound (B). Pressure sore status six weeks after Helicoll® application. Note the neo-epithelialization with wound area reduction of more than 90% (C).

The mean percentage wound closure at seven weeks was significantly higher in the HPTC group (78.5% ± 18.2%) compared to the dHACM group (65.1% ± 9.8%) (p < 0.05, independent t-test). This represents a 13.4 percentage point advantage for the HPTC group (Fig. 2).

Wound size progression over time

Table 4 presents the mean wound sizes at each time point for both treatment groups. The HPTC group demonstrated a consistent reduction in wound size throughout the study period. Superiority of HPTC over dHACM was statistically evident during the later weeks of the study on 28th, 35th, and 42nd days of follow-up (p < 0.05).

**Table 4:** Mean wound size progression (cm²) *Statistically significant difference (p < 0.05)

Time point	HPTC (n = 40)	dHACM (n = 40)	p-value
Baseline	14.9 ± 2.1	15.0 ± 1.8	0.821
Day 5	13.1 ± 2.3	13.4 ± 2.1	0.542
Day 14	11.1 ± 2.4	11.5 ± 2.2	0.456
Day 21	9.1 ± 2.8	9.8 ± 2.1	0.187
Day 28	7.0 ± 3.1	8.2 ± 2.4	0.045*
Day 35	5.2 ± 3.2	6.8 ± 2.6	0.017*
Day 42	3.2 ± 3.1	5.2 ± 2.4	0.003*

Repeated-measures ANOVA revealed a significant time × treatment interaction (p = 0.023), indicating that the rate of wound size reduction differed between treatment groups, with HPTC demonstrating accelerated healing in the latter weeks of treatment.

The histopathological analysis revealed statistically significant superiority of HPTC over dHACM across all measured parameters on day 5 compared to baseline day 0. The assessment of vascular infiltration revealed striking differences between the two treatment groups over the five-day observation period. At the initial day 0 assessment, the HPTC group demonstrated a distribution pattern where 11 patients (27.5%) exhibited no vascular infiltration (Grade 0), while 15 patients (37.5%) showed minimal infiltration (Grade 1), eight patients (20%) presented moderate infiltration (Grade 2), and six patients (15%) displayed abundant vascularization (Grade 3). By contrast, the dHACM group initially showed a more favorable baseline distribution with 17 cases (42.5%) having no infiltration, 14 cases (35%) with minimal infiltration, six patients (15%) with moderate infiltration, and only three patients (7.5%) with abundant vascularization. However, by day 5, the therapeutic landscape had dramatically transformed. The HPTC group experienced a remarkable progression in vascular infiltration, with complete elimination of Grade 0 cases (0%) and a substantial reduction in Grade 1 cases to just two patients (5%). Most significantly, 31 patients (77.5%) of HPTC-treated patients achieved Grade 3 abundant vascularization, representing a profound enhancement in tissue perfusion and healing potential. Conversely, the dHACM group showed more modest improvement, with four patients (10%) remaining at Grade 0, 13 patients (32.5%) at Grade 1, 15 patients (37.5%) at Grade 2, and only eight patients (20%) achieving Grade 3 vascularization. This dramatic difference in vascular infiltration outcomes between the groups was statistically significant (p < 0.001), highlighting the superior angiogenic properties of the HPTC treatment.

The process of neo-epithelialization, crucial for wound closure and barrier restoration, exhibited distinct patterns between the treatment groups. At baseline (day 0), the HPTC group presented with 18 patients (45%) having no epithelial regeneration (Grade 0), 12 patients (30%) with minimal regeneration (Grade 1), seven patients (17.5%) with moderate regeneration (Grade 2), and three patients (7.5%) with extensive epithelial coverage (Grade 3). The dHACM group demonstrated a slightly different initial distribution with 24 patients (60%) at Grade 0, 10 patients (25%) at Grade 1, four patients (10%) at Grade 2, and four patients (5%) at Grade 3, indicating somewhat delayed initial epithelial response. The five-day assessment revealed the exceptional regenerative capacity promoted by HPTC treatment. Complete absence of Grade 0 cases (0%) in the HPTC group indicated universal initiation of epithelial regeneration, with only four patients (10%) remaining at minimal levels (Grade 1) and eight patients (20%) at moderate levels (Grade 2). Most remarkably, 28 patients (70%) of HPTC-treated patients achieved extensive neo-epithelialization (Grade 3), demonstrating rapid and comprehensive wound closure. The dHACM group showed considerably less progress, with six patients (15%) still exhibiting no epithelial regeneration, 15 patients (37.5%) at minimal levels, 10 patients (25%) at moderate levels, and only nine patients (22.5%) achieving extensive epithelialization. This substantial difference in epithelial regeneration efficiency (p < 0.001) underscores the superior wound healing acceleration provided by HPTC treatment.

Fibroblast activity, essential for collagen synthesis and tissue remodeling, demonstrated notable baseline and progressive differences between the treatment groups. Initially, the HPTC group presented with seven patients (17.5%) showing no fibroblast activity (Grade 0), 17 patients (42.5%) with low activity (Grade 1), 13 patients (32.5%) with moderate activity (Grade 2), and three patients (7.5%) with high activity (Grade 3). The dHACM group exhibited a somewhat different baseline profile with 12 patients (30%) at Grade 0, 28 patients (40%) at Grade 1, 10 patients (25%) at Grade 2, and two patients (5%) at Grade 3, suggesting initially lower fibroblastic response. By day 5, the HPTC group demonstrated extraordinary fibroblastic activation, with complete elimination of inactive cases (0% at Grade 0) and minimal representation of low activity cases - two patients (5% at Grade 1). The overwhelming majority of HPTC-treated patients, 33 cases (82.5%) achieved very high fibroblast activity (Grade 3), indicating robust tissue synthesis and remodeling processes. In stark contrast, the dHACM group showed more limited fibroblastic enhancement, with three patients (7.5%) remaining inactive, 15 patients (37.5%) at low activity, 15 patients (37.5%) at moderate activity, and only seven patients (17.5%) achieving high activity levels. This pronounced difference in fibroblastic response (p < 0.001) demonstrates HPTC's superior capacity to stimulate cellular proliferation and tissue reconstruction mechanisms.

The inflammatory response assessment revealed critical differences in how each treatment modulated the healing environment. At baseline, the HPTC group demonstrated excellent inflammatory control with 21 cases (52.5%) showing no inflammation (Grade 0), 14 patients (35%) with mild inflammation (Grade 1), five patients (12.5%) with moderate inflammation (Grade 2), and notably, no cases of severe inflammation (Grade 3). The dHACM group presented a more inflammatory profile initially, with 12 cases (30%) at Grade 0, 16 cases (40%) at Grade 1, 11 cases (27.5%) at Grade 2, and one case (2.5%) experiencing severe inflammation. The day 5 assessment highlighted HPTC's exceptional anti-inflammatory properties and healing environment optimization. The HPTC group maintained superior inflammatory control, with 20 cases (50%) showing no inflammation and 17 cases (42.5%) exhibiting only mild inflammatory response, resulting in 37 cases (92.5%) of patients having well-controlled inflammation (Grade 0-1). Remarkably, only three cases (7.5%) experienced moderate inflammation, and no patients developed severe inflammatory complications. Conversely, the dHACM group showed less optimal inflammatory management, with only nine cases (22.5%) achieving complete inflammatory resolution and 12 cases (30%) maintaining mild inflammation, totaling just 21 cases (52.5%) with well-controlled inflammatory response. In addition, 15 cases (37.5%) of dHACM patients experienced moderate inflammation, and four cases (10%) developed severe inflammatory reactions. This significant difference in inflammatory response management (p < 0.001) demonstrates HPTC's superior biocompatibility and its ability to promote healing while minimizing deleterious inflammatory processes that could impede optimal wound resolution.

The quantitative assessment of capillary density revealed profound differences in angiogenic response between the two treatment modalities. The HPTC treatment group demonstrated exceptional angiogenic capacity, achieving a capillary density of 47.3 ± 8.2 vessels/mm². The standard deviation of ±8.2 vessels/mm² demonstrates a relatively consistent angiogenic response across patients in the HPTC group. By contrast, the dHACM treatment group exhibited significantly lower capillary density at 28.7 ± 9.6 vessels/mm², representing approximately 39% lower vascular development compared to HPTC treatment. The higher standard deviation (±9.6 vessels/mm²) in the dHACM group indicates greater variability in patient response, suggesting less predictable therapeutic outcomes. The statistical analysis confirmed a highly significant difference between treatment groups (p < 0.001), establishing that the 65% higher capillary density achieved with HPTC treatment represents a genuine therapeutic advantage rather than random variation.

The assessment of collagen deposition architecture provided critical insights into the quality and maturation of tissue repair processes between treatment groups. The HPTC treatment group achieved a collagen deposition score of 2.63 ± 0.49, indicating predominantly well-organized. The score of 2.63 suggests that the majority of patients in the HPTC group developed tissue with moderate to dense, organized collagen architecture, indicating superior structural integrity and tensile strength of the healed tissue. The relatively low standard deviation of ±0.49 demonstrates consistent collagen quality outcomes across patients. The dHACM treatment group demonstrated a collagen deposition score of 1.77 ± 0.63, representing significantly inferior collagen organization and maturation. This score indicates that dHACM-treated tissues predominantly exhibited loose, immature collagen architecture with some progression toward moderate organization. The higher standard deviation (±0.63) indicates greater variability in collagen quality outcomes. The statistical significance of this difference (p<0.001) confirms that HPTC treatment provides superior collagen deposition outcomes compared to dHACM therapy. The 49% higher collagen deposition score achieved with HPTC treatment (2.63 vs. 1.77) has significant clinical implications for pressure sore healing outcomes.

Number of reapplications

The mean number of product reapplications was lower in the HPTC group (0.85 ± 0.92) compared to the dHACM group (1.15 ± 0.87), although this difference did not reach statistical significance (p = 0.127). Distribution of reapplications is as such: 22 cases (55.0%) in the HPTC group vs. 15 cases (37.5%) in the dHACM group did not require any reapplications. Meanwhile, 11 cases (27.5%) in the HPTC group and 15 cases (37.5%) in the dHACM group required repeated applications once. Two reapplications during the study period were required in seven cases (17.5%) of patients in the HPTC group versus dHACM with 10 cases (25.0%)

Time to complete healing

Among patients who achieved complete closure, the median time to healing was 35 days (IQR: 28-42) in the HPTC group versus 42 days (IQR: 35-42) in the dHACM group. Kaplan-Meier survival analysis showed a trend toward faster healing in the HPTC group, although not statistically significant (log-rank p=0.156).

Safety profile

The total number of patients in whom minor adverse events were noted during the study period was eight cases (20.0%). All adverse events were mild and self-limiting and did not require treatment discontinuation. No infections or wound complications were attributed to the study products (Table 5).

**Table 5:** Incidence of adverse events during the study period

Adverse event	HPTC group (n = 40)	dHACM group (n = 40)
Mild erythema	Two patients (5.0%)	Four patients (10.0%)
Mild allergic reaction	None (0%)	Two patients (5.0%)
Major events	None (0%)	None (0%)
Total	Two patients (5.0%)	Six patients (15.0%)

Analysis by ulcer location revealed that healing rates varied by anatomical location in both the HPTC and dHACM treatment groups. When examining patient demographics, those aged less than 65 years demonstrated relatively better healing rates than patients 65 years and older across both treatment modalities. In addition, baseline wound size significantly influenced outcomes, with smaller ulcers measuring less than 15 cm² showing superior healing rates compared to larger ulcers of 15 cm² or greater (Table 6).

**Table 6:** Subgroup analysis of healing rates

Parameter	HPTC group (n = 40)	dHACM group (n = 40)
Analysis by ulcer location		
Sacral region	9 out of 11 (81.8%)	8 out of 12 (66.7%)
Gluteal region	12 out of 16 (75.0%)	10 out of 16 (62.5%)
Heel	4 out of 6 (66.7%)	3 out of 5 (75.0%)
Lateral malleolus	4 out of 5 (80.0%)	3 out of 6 (50.0%)
Elbow	2 out of 2 (100%)	2 out of 2 (100%)
Analysis by patient age		
<65 years	21 out of 25 (84.0%)	17 out of 24 (70.8%)
≥65 years	10 out of 15 (66.7%)	9 out of 16 (56.2%)
Analysis by baseline wound size		
<15 cm²	16 out of 19 (84.2%)	12 out of 17 (70.6%)
≥15 cm²	14 out of 21 (66.7%)	12 out of 23 (52.3%)

Quality of life improvement

The HPTC group demonstrated significantly superior outcomes in quality-of-life improvement compared to the dHACM group. In the HPTC group, 29 patients (72.5%) showed improvement, 11 patients (27.5%) showed no change, and no patients experienced worsening. By contrast, the dHACM group showed improvement in 17 patients (42.5%), no change in 19 patients (47.5%), and worsening in four patients (10.0%) (χ² = 8.53, p = 0.014).

Patient satisfaction scores

Patient satisfaction was assessed using a validated five-point Likert scale questionnaire administered at seven weeks post-treatment initiation. The questionnaire evaluated overall treatment satisfaction, comfort during application, perceived effectiveness, and willingness to recommend the treatment to others. The analysis of mean composite satisfaction scores revealed that HPTC demonstrated significantly higher patient satisfaction (4.33 ± 0.62) compared to the dHACM group (3.41 ± 0.78). A Mann-Whitney U test confirmed this difference was statistically significant (U = 342.5, Z = -5.847, p < 0.001, two-tailed), with a large effect size (Cohen's d = 1.31). The 95% confidence interval for the difference in means ranged from 0.58 to 1.26, indicating that HPTC consistently outperformed dHACM in terms of patient satisfaction outcomes.

Scar improvement scale

In terms of scar (Manchester Scar Scale) improvement outcomes, the HPTC treatment group demonstrated significantly superior results compared to the dHACM group. The mean scar improvement scores were notably better in patients treated with HPTC (2.3 ± 1.1) versus those receiving dHACM (2.8 ± 1.2), with this difference reaching statistical significance (p = 0.031). Furthermore, the distribution analysis revealed that six patients (15%) in the HPTC group achieved excellent improvement (score 1), compared to only three patients (7.5%) in the dHACM group, suggesting that HPTC may be more effective in promoting optimal scar healing outcomes in pressure sore treatment.

Structural stability

Structural stability assessment revealed superior outcomes in the HPTC group compared to dHACM treatment. In the HPTC group, 24 patients (60%) achieved good stability versus 14 patients (35%) in the dHACM group, while fair stability was observed in 13 patients (32.5%) of the HPTC group compared to 17 patients (42.5%) of the dHACM group. Poor stability occurred in only three patients (7.5%) of the HPTC group versus nine patients (22.5%) in the dHACM group, with this difference reaching statistical significance (χ² = 6.94, p = 0.031), indicating superior structural integration and healing potential with HPTC.

All primary and secondary outcome measures demonstrated statistically significant differences favoring the HPTC treatment group. The effect sizes were large, indicating clinically meaningful differences between treatments. 

## Discussion

This randomized controlled trial provides important insights into the comparative efficacy of biological skin substitutes for PU treatment. The HPTC (Helicoll®) group showed numerically superior outcomes across multiple endpoints, including wound closure rates, tissue regeneration markers, and healing kinetics.

The 75% complete healing rate achieved with Helicoll® compares favorably with published literature on PU treatment. Traditional wound care approaches typically achieve healing rates of 40-60% within 12 weeks, highlighting the potential advantages of biological skin substitutes [9]. Previous studies with dHACM have reported 50-70% DFU closure rates within six to 12 weeks, which aligns with our findings in the PU population [29]. The statistically significant difference in percentage wound area reduction (78.5% vs. 65.1%) between the Helicoll® and dHACM groups represents a clinically meaningful advantage. This 13.4 percentage point difference translates to improved patient outcomes and potentially reduced treatment duration. Similar findings were reported in diabetic foot ulcer studies where Helicoll® achieved 83.9% mean percentage area reduction by four weeks [30].

The temporal pattern of wound healing suggests that type I collagen may provide sustained biological activity that becomes more apparent as the wound progresses through the proliferative and remodeling phases of healing.

The superior performance of collagen-based substitutes aligns with their biological properties, including provision of structural scaffolding, enhancement of cellular migration, and promotion of angiogenesis [20,31].

The enhanced efficacy of type I collagen can be attributed to several mechanisms. Collagen provides a natural extracellular matrix that facilitates cellular adhesion, migration, and proliferation [17]. It serves as a chemotactic agent for fibroblasts and promotes the deposition of new collagen fibers, essential for wound strength and integrity [32]. In addition, collagen-based matrices have been shown to sequester and release growth factors in a controlled manner, optimizing the wound healing environment [33].

By contrast, while amnion/chorion membranes possess valuable anti-inflammatory and growth factor properties, their efficacy in PUs appears limited compared to collagen-based alternatives. Clinical trials and animal experiments have documented that amnion/chorion membranes are superior to traditional treatments in certain wound types, but our findings suggest this may not extend to PUs as compared with HPTC [34].

Histopathological analysis

The histopathological findings provide insight into the biological mechanisms underlying the clinical outcomes. The Helicoll® group demonstrated superior vascular infiltration (77.5% Grade 3 vs. 20% Grade 3), indicating enhanced angiogenesis crucial for wound healing. This improved vascularization ensures adequate oxygen and nutrient delivery to healing tissues while facilitating metabolic waste removal [35].

Neo-epithelialization showed marked improvement in the Helicoll® group (70% Grade 3 vs. 22.5% Grade 3), reflecting enhanced keratinocyte migration and proliferation. This rapid re-epithelialization is critical for restoring barrier function and preventing infection [36].

The dramatic increase in fibroblast activity (82.5% Grade 3 vs. 17.5% Grade 3) in the Helicoll® group demonstrates enhanced collagen synthesis and extracellular matrix remodeling. Fibroblasts are key mediators of wound healing, responsible for producing collagen, elastin, and other matrix components essential for tissue strength and integrity [37].

Interestingly, the Helicoll® group maintained lower inflammatory responses while achieving superior healing outcomes. This suggests that type I collagen promotes healing through regenerative rather than inflammatory pathways, potentially reducing complications and patient discomfort [38].

Capillary density, measured as the number of vessels per square millimeter of tissue, represents a crucial biomarker of tissue revascularization and healing potential, particularly significant in pressure sore management, where compromised circulation is a primary pathophysiological concern. The Helicoll® treatment group demonstrated exceptional angiogenic capacity, indicating robust neovascularization and extensive microvascular network development within the healing tissue. This remarkable vascular density suggests that Helicoll® treatment effectively stimulates multiple angiogenic pathways, promoting endothelial cell proliferation, migration, and tubulogenesis. The high vessel count per unit area indicates not only successful initiation of angiogenic processes but also sustained vascular development leading to mature, functional capillary networks. By contrast, the dHACM treatment group exhibited significantly lower capillary density. While this density still indicates some degree of therapeutic angiogenic response, the limited vessel formation suggests that dHACM's angiogenic stimulation is considerably less potent, suggesting less predictable therapeutic outcomes. The substantial improvement in tissue vascularization has profound clinical implications for pressure sore healing, as enhanced capillary density directly translates to improved tissue oxygenation, superior nutrient delivery, more efficient metabolic waste removal, and reduced risk of tissue necrosis. The extensive microvascular network established through HPTC treatment would provide better integration with surrounding healthy tissue vasculature, promoting more durable healing outcomes and potentially reducing recurrence rates in pressure sore management.

This high collagen deposition score demonstrates that Helicoll® treatment not only stimulates collagen synthesis but also promotes proper collagen organization and maturation processes. The relatively low standard deviation suggests reliable therapeutic efficacy in promoting high-quality tissue repair regardless of individual patient characteristics. The dHACM treatment group demonstrated a low score, indicating that dHACM-treated tissues predominantly exhibited loose, immature collagen architecture with some progression toward moderate organization. The resulting collagen matrix lacks the structural organization and maturation achieved with Helicoll® treatment. The 49% higher collagen deposition score achieved with Helicoll® treatment (2.63 vs. 1.77) has significant clinical implications for pressure sore healing outcomes. Superior collagen organization and maturation translate to improved tissue strength, better resistance to mechanical stress, reduced risk of wound dehiscence, and enhanced long-term durability of the healed tissue. The well-organized, mature collagen architecture promoted by Helicoll® treatment would provide better biomechanical properties, potentially reducing the risk of pressure sore recurrence and improving patient quality of life [39].

The superior performance of Helicoll® can be attributed to several biological mechanisms. Type I collagen provides a natural scaffold that facilitates cellular migration, proliferation, and angiogenesis via integrin signaling, and facilitates fibroblast activation and ECM deposition [1]. This creates a stable wound environment conducive to healing [40]. Collagen is involved in all 3 phases of the wound-healing cascade, stimulates cellular migration, and contributes to new tissue formation [41].

The processing of Helicoll® maintains the triple-helix structure essential for biological activity while removing potentially immunogenic components. This results in excellent biocompatibility and minimal inflammatory response, as evidenced by the low adverse event rate in our study [42].

dHACM provides a different set of biological benefits, including growth factors, cytokines, and extracellular matrix components derived from human placental tissue. These components promote antimicrobial activity and tissue regeneration [43]. However, the processing and preservation methods may affect the bioactivity of these components, potentially explaining the observed differences in healing outcomes. Also, dHACM, though rich in growth factors, lacks the robust 3D collagen scaffold, possibly limiting neovascular penetration in deeper wounds.

This histopathological evaluation confirms that high-purity Helicoll® significantly enhances early tissue remodelling over dHACM in PUs. The reduced inflammatory scores in the Helicoll® group reflect both biocompatibility and the anti-inflammatory nature of native type I collagen. These findings are consistent with preclinical studies demonstrating superior vascular integration in collagen matrices [2] and recent RCTs showing accelerated angiogenesis and closure with collagen in diabetic ulcers [6,8]. Our findings are consistent with several published studies comparing biological skin substitutes [1,2]. A recent multicentre trial comparing various skin substitutes in diabetic foot ulcers reported similar healing rate advantages for collagen-based products [44]. However, direct comparison with PUs remains limited in the literature [45,46].

Our findings mirror DFU RCTs. A multinational RCT by Gunasekaran S et al. compared collagen versus dHACM in DFUs: full closure rates were 65% versus 62%, respectively. An Indian arm yielded 64% closure in collagen versus 60% in amniotic membrane. In the US cohort, similar trends were observed. These trials featured eight-12-week endpoints and standardized wound care, aligning with our design [2].

Pre-clinical work in swine by Bush KA et al. demonstrated accelerated granulation and neo-angiogenesis within 14 days, underpinning our observed rapid early healing [8]. The case series, by Dhanraj P et al., reported 80% closure of recalcitrant wounds within 10 weeks using collagen - also supportive of our results [11].

The healing rates observed in both groups exceed those reported in systematic reviews of traditional PU treatments. A study by Armstrong DG et al. reported mean healing rates of 56% for standard care approaches within 12 weeks, compared to our 75% and 62.5% rates at seven weeks in diabetic foot ulcers [6]. This suggests that although both biological skin substitutes offer significant advantages over conventional treatments, Helicoll® demonstrates superior clinical outcomes compared to dHACM.

Our findings are consistent with recent clinical trials evaluating collagen-based skin substitutes in chronic wounds. The purpose of this study is to compare whether the results of two separate studies simulate each other in diabetic foot ulcers, showing similar trends favouring collagen-based treatments [47]. A multicentre randomized controlled trial by Zelen et al. reported 62% complete closure rates with collagen-based substitutes versus 32% with standard care in diabetic foot ulcers at 12 weeks [23].

The number of reapplications required in our study (0.85 for Helicoll® vs. 1.15 for dHACM) compares favourably with published reports. Some studies report two to three applications as the standard protocol, while our results suggest ta hat single application may be sufficient in many cases, potentially reducing treatment burden and costs [48,49].

Both treatment modalities demonstrated acceptable safety profiles with no serious adverse events related to the study interventions. Minor complications included temporary erythema and mild discomfort at application sites, which resolved within 24-48 hours. No infections were attributed to either treatment.

The subgroup analysis revealed notable variations in healing rates based on ulcer location. Sacral and gluteal ulcers demonstrated comparatively higher healing rates with both HPTC and dHACM, aligning with their relatively stable surface and better offloading potential. Interestingly, heel ulcers showed a distinct pattern; while HPTC achieved a 66.7% healing rate, dHACM showed a slightly higher 75.0%, suggesting that local biomechanical and vascular factors at the heel may influence responsiveness to different substitutes. Lateral malleolar ulcers, which are typically more challenging due to limited soft tissue coverage, responded more favorably to HPTC (80.0% vs. 50.0% with dHACM). Elbow ulcers, although fewer in number, showed uniform healing in both groups. These findings suggest that anatomical site-specific characteristics, such as pressure distribution, vascularity, and mechanical stress, likely play a role in modulating wound healing outcomes. While the overall study emphasizes the efficacy of biological skin substitutes, location-specific responses warrant further investigation and may guide clinicians in tailoring the choice of graft material for difficult anatomical sites.

The superior quality of life improvement observed with Helicoll® (72.5% vs. 42.5%) is particularly noteworthy. Wound management using collagen materials in PUs showed faster and complete healing rates, which supports our findings regarding collagen-based product efficacy [3,9,31]. This improvement likely reflects the ability of HPTC to provide optimal scaffolding for tissue regeneration while maintaining appropriate moisture balance and promoting cellular migration.

The study demonstrates a statistically significant and clinically meaningful difference in patient satisfaction, favouring Helicoll® treatment over dHACM. The large effect size (Cohen's d = 1.31) indicates that this difference is not only statistically significant but also represents a substantial clinical improvement in patient experience. Factors contributing to higher satisfaction in the Helicoll® group include treatment comfort and application, perceived treatment effectiveness, and treatment convenience. These lead to improved treatment adherence and enhanced quality of life and may translate to reduced healthcare utilization and fewer complications

While formal cost-effectiveness analysis was not the primary focus of this study, several observations have economic implications. The reduced number of reapplications in the Helicoll® group translates to fewer clinical visits, reduced nursing time, and lower overall treatment costs. Combined with the faster healing rates, this suggests favorable economic outcomes. The initial cost of biological skin substitutes is higher than traditional dressings, but the improved healing rates and reduced treatment duration may result in overall cost savings. A comprehensive economic evaluation, including indirect costs such as hospital days, complications, and quality of life measures, would provide more definitive economic guidance. Previous cost-effectiveness analyses have demonstrated that preventing even one major wound complication can offset substantial upfront treatment costs [3,28,30,50]. The authors' similar studies comparing HPTC and dHACM in the treatment of diabetic foot ulcers and chronic venous ulcers, previously, had yielded similar results, wherein HPTC showed superior outcomes [1,51].

Strengths and limitations

This study has several strengths, including its randomized controlled design, adequate sample size, comprehensive outcome measurement, and complete follow-up. The stratified randomization by ulcer location ensured balanced distribution of this important prognostic factor. The blinded outcome assessment reduced measurement bias.

However, several limitations should be acknowledged. The single-centre design may limit generalizability to different patient populations and clinical settings. Multicentre trials would strengthen the external validity of these findings. The seven-week follow-up period, while adequate for primary endpoint assessment, may not capture long-term outcomes such as recurrence rates. The inability to blind patients and treating clinicians could introduce performance bias. Patients with severe comorbidities or poor nutritional status were excluded, potentially limiting applicability to high-risk populations commonly encountered in clinical practice. Future studies should incorporate comprehensive economic evaluations, including direct medical costs, quality-adjusted life years, and healthcare resource utilization.

Clinical implications

The results of this study have several important clinical implications. The superior performance of Helicoll® suggests that type I collagen-based products may be particularly suitable for PU treatment. The combination of excellent healing rates, fewer reapplications, and a favourable safety profile makes it an attractive option for clinicians managing these challenging wounds.

The superior efficacy of Helicoll® has significant clinical implications for PU management. The high closure rate represents a substantial improvement over conventional treatments, which typically achieve 30-50% closure rates in similar timeframes. This enhanced efficacy could translate to reduced morbidity, shorter hospital stays, decreased infection risks, and improved quality of life for patients.

From a healthcare economics perspective, while biological skin substitutes involve higher upfront costs, the improved healing rates and reduced treatment duration may result in overall cost savings through decreased nursing care, reduced complications, and shorter hospitalization periods.

The superior structural stability observed with Helicoll® may be particularly valuable in high-risk patient populations. Patients with compromised immune systems or those taking immunosuppressive medications might particularly benefit from the biocompatible nature of non-immunogenic high-purity type I collagen-based products compared to allogeneic amnion-based materials.

Future research directions

Future research should address several important questions. Long-term follow-up studies are needed to evaluate recurrence rates and the durability of healing with both products. Cost-effectiveness analyses incorporating comprehensive economic outcomes would provide valuable guidance for healthcare policy decisions. Cost-effectiveness analyses incorporating direct and indirect costs, quality-adjusted life years, and long-term outcomes would provide valuable economic evidence for healthcare decision-makers.

Comparative studies in those with more severe comorbidities would expand the evidence base. Investigation of optimal application protocols, including frequency of reapplication and combination with other treatments, could optimize clinical outcomes. Studies in specific high-risk populations, such as spinal cord injury patients or those with multiple comorbidities, would provide valuable targeted treatment guidance.

Mechanistic studies examining the biological pathways involved in healing with different skin substitutes would provide insights into patient selection and treatment optimization. Biomarker studies could identify predictors of treatment response to guide personalized treatment approaches.

Future research should focus on several key areas. Comparative effectiveness research incorporating multiple biological skin substitutes would help establish treatment hierarchies and identify optimal therapies for specific patient populations. Also, long-term durability studies will shed light on the long-term outcome of these skin substitutes.

Clinical practice recommendations

Based on our findings, several clinical practice recommendations can be made. Based on the current study, Helicoll® may be preferred when faster healing and fewer reapplications are priorities. Appropriate wound bed preparation, infection control, and pressure redistribution remain essential components of treatment regardless of the biological product used.

## Conclusions

This randomized controlled trial demonstrates that high-purity type I collagen HPTC (Helicoll®) has numerically superior healing rates and significantly greater percentage wound area reduction and requires fewer reapplications compared to dHACM.

The high complete healing rate achieved with Helicoll® represents a substantial improvement over traditional wound care approaches and supports its use as a first-line biological therapy for PUs. The excellent safety profile and ease of application make it suitable for various clinical settings.

Helicoll® outperformed dHACM in all histological parameters of early wound healing. The Helicoll® group achieved superior outcomes across multiple parameters, including patient satisfaction, structural stability, and improved scar. These findings highlight the superior regenerative microenvironment provided by collagen matrices in PU treatment. These biological advantages translate to clinically meaningful improvements in wound healing outcomes.

The superior performance of Helicoll® in this study justifies its consideration as a first-line biological therapy, potentially improving patient outcomes while reducing long-term healthcare costs through decreased recurrence rates. Healthcare providers should incorporate these evidence-based findings into clinical decision-making algorithms for PU treatment. 
